# Is Opioid-free Anesthesia Possible by Using Erector Spinae Plane Block in Spinal Surgery?

**DOI:** 10.7759/cureus.18666

**Published:** 2021-10-11

**Authors:** Yasin Taşkaldıran

**Affiliations:** 1 Anesthesiology and Reanimation, Erzurum Regional Training and Research Hospital, Erzurum, TUR

**Keywords:** erector spinae plane block, lumbar herniated disc surgery, postoperative analgesia, intraoperative analgesia, opioid consumption

## Abstract

Objective: Erector spinae plane (ESP) block can be a method to be used for postoperative pain control in lumbar herniated disc operations. The aim of this study is to investigate the effect of erector spinae block in lumbar herniated disc operation on intraoperative and postoperative opioid consumption.

Methods: Sixty patients scheduled for lumbar herniated disc surgery were included in the study. Patients were randomized into two groups: ESP block and control. Ultrasound-guided ESP block with 20 ml 0.25% bupivacaine at the bilateral L3 vertebral level was applied preoperatively to all patients in the ESP group. Patients in both groups were provided with intravenous patient-controlled analgesia (PCA) device containing fentanyl for postoperative analgesia. Fentanyl consumption and visual analogue scale (VAS) score were recorded at 15 min, 1, 6, 12, and 24 hours postoperatively.

Results: Fentanyl consumption (group C: 59.3 ± 20.66, group E: 41.3 ± 21.61, p: 0,02) and VAS score (group C: VASm 4 (2-4), group E: 2 (2-4), p: 0.009) decreased with ESP block application at postoperative one hour. No difference was detected between the two groups in terms of fentanyl consumption and VAS score at 6, 12, and 24 hours postoperatively (p>0.05). The intraoperative heart rate of patients in the ESP group was lower than the control group (p<0.05).

Conclusion: ESP block decreases opioid consumption and VAS score at postoperative one hour in patients, and also patients who receive ESP block do not require intraoperative opioid administration.

## Introduction

Non-surgical treatment methods are performed on the majority of patients suffering from back pain due to the lumbar herniated disc. However, for the remaining patients, the surgical method is preferred, and the number of patients requiring operation increases. In 2003, 2.2 of every 1000 hospital admissions were patients to be operated on due to lumbar disk disease [1]. Moreover, in the last decade, the number of operations due to lumbar degenerative disc disease increased by 2.4-fold [2]. In these frequently performed surgeries, it was found that the median visual analogue scale (VAS) scores at the first postoperative 24 hours were five in patients who underwent general anesthesia [3]. For postoperative pain control, the use of multimodal analgesia is recommended [4]. Regional anesthesia may play an important role in multimodal analgesia, but its use in spinal surgeries is not sufficiently common [5]. Opioids are the primary drugs used for analgesia. Opioid use and related side effects can be reduced with regional anesthesia and other methods [4].

Erector spinae plane (ESP) block can be a method to be used for postoperative pain control in lumbar herniated disc operations. In this method, a local anesthetic is applied between erector spinae muscles and transverse processes of the vertebra. On the other hand, local anesthetics exert their effect by infiltrating the ventral and dorsal rami of the spinal nerve. In MR images, it was found that local anesthetic also infiltrates the epidural space and sympathetic chain [6-8]. There are studies that ESP block can be used for pain control after lumbar vertebra operations [9]. While there are differences between the duration of effect of the ESP block, there is not enough assessment about its effects on the intraoperative period.

The primary aim of the study is to evaluate opioid consumption in the postoperative period. Its secondary purpose is to evaluate the intraoperative effect of the ESP block.

## Materials and methods

The study was performed after obtaining the approval of the local ethics committee (Ethics Committee No. 2020/06-74) and the Ministry of Health Medicines and Medical Devices Agency (Code: 20-AKD-01). The study was registered with anzctr.org.au (ACTRN12619001763134p).

After the patients’ consent were obtained, they were enrolled in the study. A total of 62 patients aged between 18 and 75 years, classified as American Society of Anesthesiologists (ASA) I-II, and scheduled for the elective lumbar herniated disc were enrolled in the study. Patients in emergency cases, pregnant and/or lactating, coagulopathic, using antiaggregant and anticoagulant drugs, a history of vertebral surgery, diagnosed with malignancy, or with scheduled instrumentation and opioid usage or tolerance were excluded from the study. Two patients were excluded from the study because the block was unsuccessful. Patient groups were determined in a randomized manner, using a computer software. Patients were divided into two groups as group E (ESP block group) and group C (control group).

After the patients were divided into groups, 0.01 mg/kg midazolam was administered intravenously. Then, the patients were transferred to the operating room. The patients’ pulse oximetry and electrocardiogram signals were monitored and their blood pressures were measured using noninvasive methods.

To induce general anesthesia, propofol (2-3 mg/kg), fentanyl (1 mcg/kg), rocuronium (0.6 mg/kg), and lidocaine (1 mg/kg) were administered intravenously to both groups. For the maintenance of general anesthesia, sevoflurane (1-2%), oxygen (40%), and air (60%) were used.

Povidone-iodine 10% was applied to the lumbar area of the patients receiving group E. Ultrasound probe was covered with a sterile sleeve. Blocks were applied in-plane using a 21-gauge 10-cm needle (Braun Sonoplex, Melsungen, Germany), via an ultrasound-guided linear probe (Esaote MyLab™ 30 US machine, Esaote SpA, Florence, Italy, 6-15 MHz). The linear probe was sagitally placed 2-3 cm lateral to the spinous process of the L3 vertebrae. The needle was inserted from cranial to caudal direction. By injecting 1-2 ml of 0.9% physiological saline solution into the interfacial plane between erector spinae muscle and transverse process, the location of the tip was confirmed. Then, 20 ml of 0.25% bupivacaine was administered and the block was also applied to the other side of the vertebrae. Hemilaminectomy was performed in patients with a surgical incision in the midline. No additional medication was applied to the surgical area by the surgeon. The incision was performed 10 minutes after applying the block. In the case of an increase of more than 20% in blood pressure or heart rate after the surgery started, the applied block was considered failed. For this assessment, blood pressure and heart rate measurements taken 10 minutes after applying the block were taken as a reference. In the case of failed blocks, the patients were given an intravenous infusion of remifentanil (0.1-1.0 mcg/kg/min), intravenous paracetamol (1 g), and tramadol (1 mg/kg) were administered for postoperative analgesia and the patients were removed from the study. In patients with successful blocks, no additional analgesia was applied intraoperatively and postoperative.

For intraoperative analgesia, group C was given intravenous remifentanil infusion (0.25-1.0 mcg/kg/min). For postoperative analgesia, intravenous paracetamol (1 g) and tramadol (1 mg/kg) were administered 30 minutes before the termination of the operation. Prior to extubation, both groups were given sugammadex (2 mg/kg).

Postoperatively, the patients were monitored in the post-anesthesia care unit for one hour, and then they were followed up in the service. For postoperative analgesia, patient-controlled analgesia (PCA) was administered. PCA contained fentanyl and the variables were set as follows: drug concentration: 10 mcq/ml, demand: 20 mcg, lockout: 5 min, maximum four-hour dose: 300 mcg, loading dose: none, infusion: none. During patient follow-ups, 75 mg diclofenac Na was administered intramuscularly to those with NRS>4/10.

The patients’ resting visual analogue scale (VASr) scores and movement visual analogue scale (VASm) scores were measured at 15 min, 1 h, 6 h, 12 h, and 24 h. Moreover, the patients’ fentanyl consumption and analgesia requirements were monitored for 24 hours. These were performed by pain management nurses who did not know which group they were considering. Data were collected prospectively.

Our sample size was calculated based on previous studies [10]. According to this, assuming a power of 90%, alpha value of 0.05 and d value of 0.85, the calculated sample size was 30 patients per group.

All statistical analyses were performed using IBM SPSS for Windows® version 20.0 software (SPSS, Chicago, IL, USA). The Kolmogorov-Smirnov test was used to determine normality of data distribution. Univariate analysis compared in groups means using a two-sample, independent t-test assuming equal variances for continuous variables. For data without normal distribution, the Mann Whitney U test was performed. Ratios were compared using Chi-Square. Categorical variables were compared using Fisher exact test. A p-value <0.05 was considered statistically significant.

## Results

**Figure 1:**
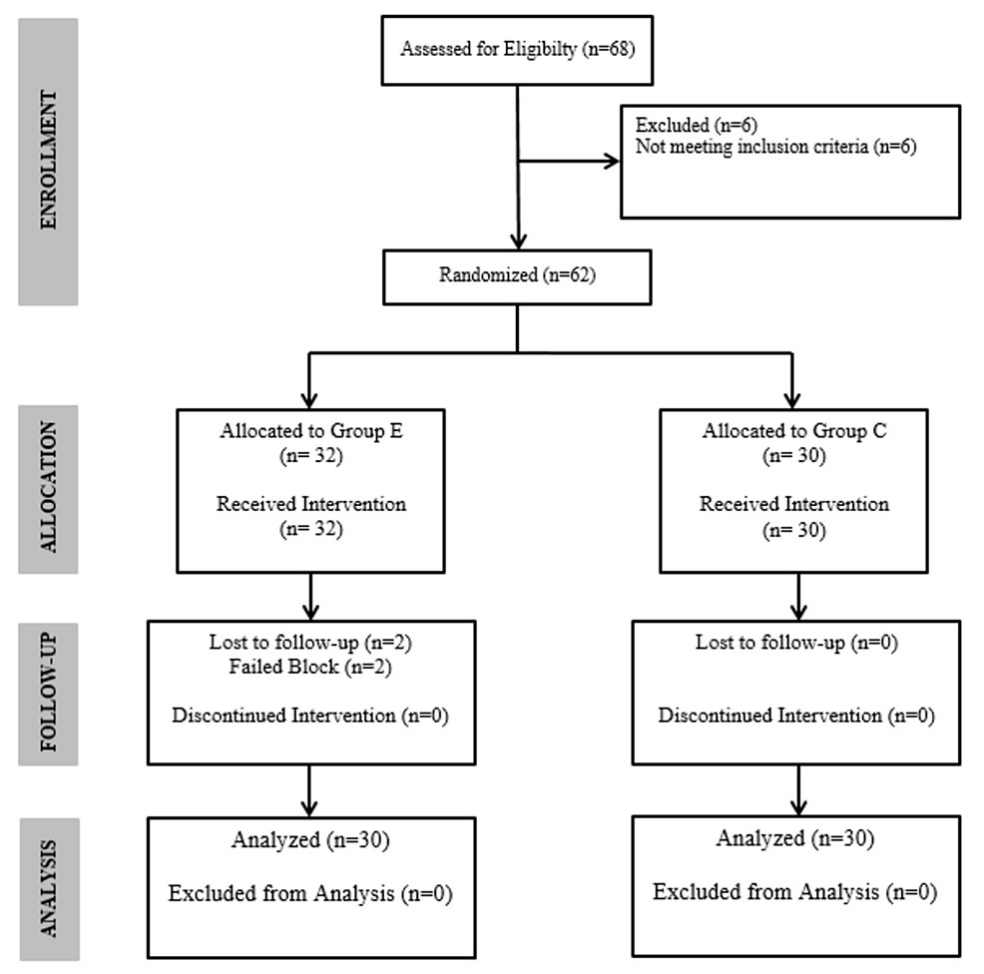
Consort flow diagram

A total of 60 patients were enrolled in the study (Figure 1). In the study, no difference was detected between the groups in terms of demographic data. It was also found that the patients included in the study were not different in terms of ASA class, height, weight, BMI, duration of surgery, length of the surgical incision, and the number of discs operated. It was found that the duration of anesthesia was longer in patients in group E (p<0.05; Table 1).

**Table 1:** Patient demographics and operation characteristics. Data are presented as mean ± standard deviation or number. Group C, control group; group E, erector spinae plane block group; BMI: body mass index; F: female; M: male.

	Group C (n=30)	Group E (n=30)	p-Value
Age (year)	53.3 ± 9.9	51.4 ± 13.05	0.529
Sex F/M	14/16	15/15	0.769
ASA I/II	7/23	6/24	0.754
Weight (kg)	73.6 ±7.94	74.5 ± 9.36	0.69
Height (cm)	170 ± 6.54	163.9 ± 31.63	0.307
BMI (kg/m^2^)	25.4 ± 1.84	25.9 ± 2.64	0.386
Anesthesia time (min)	84.6 ± 14.01	92.8 ± 15.8	0.039
Surgery time (min)	69.8 ± 12.69	64.6 ± 15.75	0.167
Incision length (cm)	7 ± 1.57	7.5 ± 2.16	0.378
Number of operated lumbar discs (1/2)	22/8	24/6	0.542

**Figure 2:**
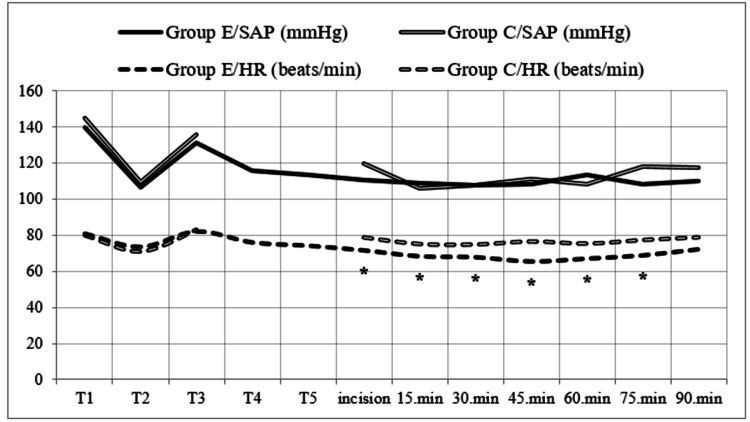
Vital signs of the two groups. *p<0.05 Heart rate: Comparison of group C and group E. T1: first measurement; T2: after induction; T3: after intubation; T4: after block; T5: 10 minutes after the block; Group C: control group; Group E: erector spinae plane block group; HR: heart rate; SAP: systolic arterial pressure.

Considering the vital values of the patients in group E, the patients did not need additional opioids. Therefore, while remifentanil was not given to the patients in group E, remifentanil was administered to group C in the range of 0.25-1.0 mcg/kg/min. During the follow-up of vital signs, there was no difference between the patients in terms of systolic arterial pressure. In heart rate follow-ups, it was found that the heart rate of patients in group E was lower (p<0.05). Both groups were similar in terms of their pre-incision heart rates (Figure 2).

It was found that VASr and VASm scores were lower at 15 minutes and 1 hour in group E (p<0.05). No difference was detected between the groups in terms of 6 hours, 12 hours, and 24 hours VASr and VASm scores (Table 2).

**Table 2:** Comparison of the two groups in terms of VAS pain scores. *p<0.05. Data are presented as median (interquartile range). Group C: control group; Group E: erector spinae plane block group; VASr: resting visual analogue scale score; VASm, movement visual analogue scale score.

	VASr			VASm		
	Group C (n=30)	Group E (n=30)	p-Value	Group C (n=30)	Group E (n=30)	p-Value
15th min	4 (2–6)	2 (0–4)	0.002*	6 (4–6)	2 (2–4)	0.000*
1st hour	2 (2–2.5)	2 (0–2)	0.014*	4 (2–4)	2 (2–4)	0.009*
6th hour	2 (2–2)	2 (0–2)	0.055	2 (2–4)	2 (2–4)	0.364
12th hour	2 (1.5–2)	2 (0–2)	0.138	2 (2–4)	2 (2–4)	0.476
24th hour	2 (0–2)	1 (0–2)	0.194	2 (2–2)	2 (2–2)	0.617

In terms of opioid consumption, it was found that fentanyl consumption in the first one hour was less in group E (p<0.05). Both groups were similar in terms of their fentanyl consumption between the 1st and 24th hours. Total fentanyl consumption was the same in both groups (Table 3).

**Table 3:** Fentanyl consumption delivered by patient-controlled analgesia postoperatively. *p<0.05. Data are presented as mean ± standard deviation. Group C: control group; Group E: erector spinae plane block group.

	Fentanyl consumption (μg)			Demand number		
	Group C (n=30)	Group E (n=30)	p-Value	Group C (n=30)	Group E (n=30)	p-Value
0–1 hour	59.3 ± 20.66	41.3 ± 21.61	0.02*	4.6 ± 1.73	2.4 ± 1.9	0.000*
1–6 hour	107.3 ± 44.09	103.3 ± 81.38	0.814	7.4 ± 3.41	6.1 ± 5.13	0.253
6–12 hour	110.6 ± 34.33	123.3 ± 70.48	0.38	7.3 ± 3.09	6.9 ± 4.1	0.646
12–24 hour	128.6 ± 65.74	134 ± 85.52	0.788	7.6 ± 3.8	7.3 ± 4.72	0.765
Total	406 ± 130.79	402 ± 184.22	0.923	27.1 ± 9.02	22.9 ± 11.09	0.113

## Discussion

In our study, we found that a decrease was observed in opioid consumption and VASr and VASm scores within the first hour after surgery. We observed that ESP block did not have an effect on opioid consumption and VASr and VASm scores between the 1st and 24th hours after surgery. Moreover, ESP block proved to be sufficiently effective as intraoperative analgesia. Monitoring vital signs revealed that heart rate was lower but blood pressure levels remained the same in the ESP block group.

Many studies have shown that postoperative opioid consumption can be reduced by ESP block [11]. It is recommended to use local anesthetic techniques to reduce perioperative opioid consumption in ERAS (enhanced recovery after surgery) protocols. For this purpose, there are many studies related to ESP block for postoperative opioid consumption. However, there are not enough studies on the effect of ESP block on intraoperative opioid consumption. According to the ERAS protocols, the reduction in opioid consumption of the patients provides benefit in the reduction in postoperative opioid-related complications and in the duration of hospital stay [12]. In our study, postoperative effects of ESP block, as well as intraoperative effects, were evaluated. Opioids were not used in the intraoperative period due to the ESP block applied to the patients before the incision. In this respect, we think that ESP block contributes to a decrease in opioid need in the perioperative period. In addition, we think that ESP block can be used for intraoperative analgesia as an alternative to opioids in such operations.

In the literature, ESP block was reported to reduce the pain level. However, the duration of this effect differs among the studies. Besides, the types of opioids used for postoperative analgesia also differ among the studies. While Yayık et al. reported the duration of effect of the ESP block as 24 hours, they used tramadol for postoperative analgesia [13]. On the other hand, Singh et al. used morphine in their study and reported the duration of effect as six hours [14]. In our study, fentanyl was used as a postoperative analgesic and its duration of effect was found to be one hour. In addition, patients did not need additional opioids and analgesics in the intraoperative and postoperative periods. Differences in the efficacy of the ESP block may be attributed to the different opioids used for postoperative analgesia. Fentanyl can have a stronger analgesic effect than other opioids [15]. Due to this characteristic, fentanyl might have obscured the efficacy of ESP block and resulted in the detection of a shorter duration of effect in our study.

ESP block showed its effect in patients for an average of 2.5 hours. This period constitutes the intraoperative and postoperative first hour. In the intraoperative period, no additional analgesia was administered to the patients. VAS scores and opioid consumption of the patients in group E were lower in the postoperative first hour. In addition, the effect of ESP block decreased after the first hour postoperatively. In this effect of ESP block, bupivacaine may have been effective in the metabolism of HCl in the body. The t1/2 duration of bupivacaine HCl administered from the epidural is 2.7 hours [16]. In addition, bupivacaine HCl applied in ESP block passes into circulation faster than epidural [17]. The periods of loss of effect and metabolism of bupivacaine are consistent. The pharmacokinetics of bupivacaine may have been effective in the similarity between the results of group E and group C after the postoperative first hour.

High doses of opioid use in the intraoperative period may contribute to the development of postoperative hyperalgesia, leading to an increase in pain scores. Remifentanil plays a major role in the development of hyperalgesia. Administration of remifentanil infusion to patients for 30 minutes may cause hyperalgesia development [18]. Administration of more than 0.1 mcg/kg/min remifentanil may lead to opioid-induced hyperalgesia and opioid tolerance. To develop opioid-induced acute tolerance, at least 0.25 mcg/kg/min, and to develop opioid-induced hyperalgesia, at least 0.2 mcg/kg/min remifentanil must be administered [19]. Intraoperative use of opioids at a high dose leads to an increase in morphine use in the first two postoperative hours. Moreover, it is believed to cause an increase in pain scores between postoperative 2 and 24 hours [20]. In our study, remifentanil was used intraoperatively in the control group and fentanyl consumption at the postoperative one hour was higher. In LDH operations, the use of ESP block instead of opioids in the intraoperative period might lead to a decrease in postoperative hyperalgesia and side effects due to opioid consumption.

There can be differences between the area of application of local anesthetic and maximum blood concentration (Cmax). When 400 mg lidocaine was used, the Cmax value in femoral and sciatic nerve block was 2625±610 ng/mL [21] and it was 3600±700 ng/ml in transversus abdominis plane block [22]. In ESP block which used an average of 236 mg lidocaine, Cmax was 2590 ng/mL [17]. Although lower doses were used in the ESP block, similar Cmax levels were attained. This might pose a risk when the side effects of the drugs used in ESP block are observed. Bupivacaine used in our study has a negative inotropic effect [23]. There is a lack of studies on blood drug concentrations when bupivacaine is used in ESP block. When 100 mg bupivacaine was used, Cmax was 1022±253 ng/ml in axillary brachial plexus block [24] and Cmax was 802.36 ng/mL in transversus abdominis plane block [25]. Considering the study on ESP block using lidocaine, the Cmax value of bupivacaine to be used in ESP block can be higher. In the study on canine models, in plasma bupivacaine concentrations below 1500 ng/ml, it was found that the duration of conduction via the His-Purkinje system and ventricle was longer; and sinoatrial node and atrioventricular node were suppressed in plasma bupivacaine levels above 1500 ng/ml [26]. Since the bioavailability of the ESP block is much higher than other areas of application, the risk of a cardiac negative inotropic effect is much higher. The lower heart rate in group E in our study could be associated with blood bupivacaine levels.

The groups were different in terms of their anesthesia durations. This difference was due to the time elapsed between the induction of anesthesia and the application of ESP block. Application of ESP block when the patient is under anesthesia provides patient comfort. Since VAS scores and fentanyl consumption were lower in patients who received ESP block, we believe that the length of anesthesia does not have any negative effects on the study.

The study has several limitations. First, the study was an observational study. Second, since the block was applied after the patient was anesthetized, block interval could not be measured.

## Conclusions

In conclusion, the application of the ESP block decreases opioid consumption at postoperative one hour and VAS scores in patients who underwent lumbar herniated disc operation. Moreover, patients who receive ESP block do not require additional opioids in the intraoperative period. Opioid consumption can be reduced by using ESP block in the perioperative period.
